# In Vitro Investigation of Thiolated Chitosan Derivatives as Mucoadhesive Coating Materials for Solid Lipid Nanoparticles

**DOI:** 10.1021/acs.biomac.1c00776

**Published:** 2021-08-30

**Authors:** Richard Wibel, Doris E. Braun, Laurenz Hämmerle, Arne M. Jörgensen, Patrick Knoll, Willi Salvenmoser, Christian Steinbring, Andreas Bernkop-Schnürch

**Affiliations:** † Department of Pharmaceutical Technology, University of Innsbruck, Institute of Pharmacy, Center for Chemistry and Biomedicine, 6020 Innsbruck, Austria; ‡ Department of Zoology, University of Innsbruck, Technikerstr. 25, 6020 Innsbruck, Austria

## Abstract

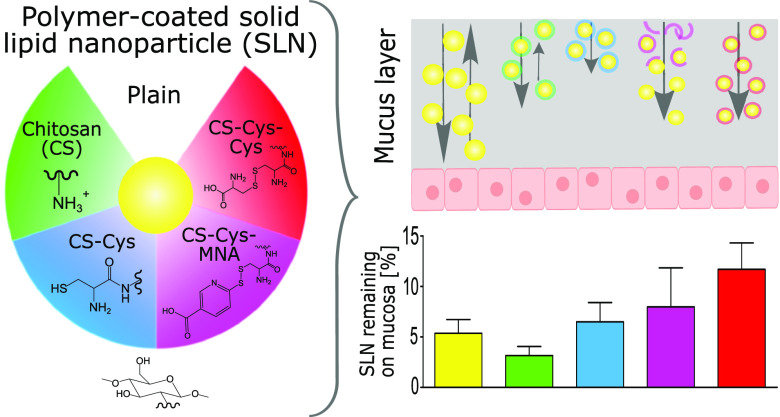

In the present study, chitosan (CS) was thiolated by introducing l-cysteine via amide bond formation. Free thiol groups were protected with highly reactive 6-mercaptonicotinic acid (6-MNA) and less-reactive l-cysteine, respectively, via thiol/disulfide-exchange reactions. Unmodified CS, l-cysteine-modified thiolated CS (CS-Cys), 6-MNA-S-protected thiolated CS (CS-Cys-MNA), and l-cysteine-S-protected thiolated CS (CS-Cys-Cys) were applied as coating materials to solid lipid nanoparticles (SLN). The strength of mucus interaction followed the rank order plain < CS < CS-Cys-Cys < CS-Cys < CS-Cys-MNA, whereas mucus diffusion followed the rank order CS-Cys < CS-Cys-Cys < CS < CS-Cys-MNA < plain. In accordance with lower reactivity, CS-Cys-Cys-coated SLN were immobilized to a lower extent than CS-Cys-coated SLN, while CS-Cys-MNA-coated SLN dissociated from their coating material resulting in a similar diffusion behavior as plain SLN. Consequently, CS-Cys-Cys-coated SLN and CS-Cys-MNA-coated SLN showed the highest retention on porcine intestinal mucosa by enabling a synergism of efficient mucus diffusion and strong mucoadhesion.

## 1 Introduction

To overcome hurdles such as poor solubility of hydrophobic drugs, presystemic metabolism, and poor absorption, lipid-based formulations (LBF) are widely investigated as mucosal drug delivery systems.^1−3^ To improve the efficacy of these formulations, several researchers applied polymeric coatings that, among other characteristics, enable enhanced mucoadhesion and, thus, prolonged residence time at mucosal sites.^4,5^ CS can be considered as one of the most thoroughly investigated polymeric coating materials.^4,6^ CS itself, as well as its modifications, has been applied to various LBF such as liposomes,^7,8^ nanostructured lipid carriers,^9^ and SLN.^4,6^

Because of cationic amino groups, CS undergoes ionic interactions with carboxylic moieties of mucins,^8,10^ the main component of mucus layers. Introducing thiol groups on CS is a well-established strategy to increase mucoadhesive properties further.^11−13^ Thiolated polymers form disulfide bonds with thiol moieties of mucins and thereby improve mucoadhesive properties compared to solely ionic interactions. Beyond mucoadhesion, thiolation enables several promising properties such as permeation enhancement, efflux pump, and enzyme inhibition.^14^

First, thiolated polymers exhibited free thiol groups that were stable as a powder and tablet^15^ but susceptible to oxidation after swelling in water.^16^ To increase stability toward oxidation and to improve the reactivity of thiol moieties, 6-MNA was proposed as a highly reactive S-protecting ligand.^17,18^ Recently, researchers introduced less-reactive S-protecting ligands such as l-cysteine and *N*-acetylcysteine. As a result of their comparatively lower reactivity, they enable diffusion into the mucus layer before forming disulfide bonds.^19,20^ Thus, S-protection with l-cysteine allows thiolated polymers to reach deeper into the mucus and surpass the loose outer mucus layer that undergoes rapid turnover.

So far, however, the potential of this type of S-protected CS has not been evaluated as a polymeric coating material for LBF. It was, therefore, the aim of this study to design and characterize such delivery systems. CS was thiolated and, subsequently, free thiol groups were protected with 6-MNA and l-cysteine, respectively, by means of thiol/disulfide-exchange reactions. These thiolated polymers were applied as coating materials to SLN consisting of cetyl palmitate as a solid lipid matrix, Pluronic F127 as a surfactant, and egg lecithin as a co-surfactant. The resulting SLN formulations were then subjected to in vitro investigation of stability and mucus-interacting properties.

## 2 Materials and Methods

### 2.1 Materials

Low-molecular-weight CS (50–190 kDa), 6-MNA (technical grade, 90%), *N*-hydroxysuccinimide (NHS) (98%), l-cysteine (≥97%), l-cysteine hydrochloride monohydrate (l-cysteine-HCl × H_2_O) (≥98%), 6,6′-dithiodinicotinic acid (6,6′-DTNA) (technical grade, 85%), fluorescein isothiocyanate (FITC) (≥90%), L-glutathione (reduced, ≥99%), 5,5′-dithiobis(2-nitrobenzoic acid) (DTNB) (≥98%), pepsin from porcine gastric mucosa (≥250 U/mg), potassium dihydrogen phosphate (KH_2_PO_4_) (99.5–100.5%, Ph. Eur.), pancreatin from porcine pancreas (4× USP specification), potassium chloride (KCl) (for analysis), protease inhibitors, sodium borohydride (≥98%), and sodium phosphate monobasic (Na_2_HPO_4_) (Ph. Eur, USP) were purchased from Sigma-Aldrich (Vienna, Austria). Egg lecithin (Lipoid E80) was a kind gift from Lipoid GmbH (Ludwigshafen, Germany). Pluronic F127 and Lumogen Red (LGR) were donated from BASF (Ludwigshafen, Germany). Sodium chloride (99.5%), *N*,*N*-dimethylformamide (DMF) (≥99%), and cetyl palmitate (98%) were products from Acros (Geel, Belgium). Acetic acid (100%, Ph. Eur.), 1-ethyl-3-(3′-dimethylaminopropyl)carbodiimide hydrochloride (EDAC × HCl) (≥99%), dimethyl sulfoxide (DMSO) (≥99.5%), hydrochloric acid (HCl) (37%), and sodium hydroxide (NaOH) pellets were purchased from Roth (Karlsruhe, Germany). FaSSIF/FeSSIF/FaSSGF powder was purchased from Biorelevant (London, U.K.).

### 2.2 Methods

#### 2.2.1 Synthesis of CS-Cys

To activate carboxylic moieties of l-cysteine, 1.50 g of l-cysteine, 0.60 g of EDAC × HCl, and 0.36 g of NHS were stirred for 3 h in DMF. Subsequently, 0.50 g of low-molecular-weight CS was dissolved in 200 mL of demineralized water and the pH was adjusted to 2 using 1 M HCl. Activated l-cysteine was added to the CS solution and pH was adjusted to 5.5 using 1 M NaOH. After 1 h of stirring at room temperature, the mixture was transferred to a dialysis tube with a molecular weight cut-off of 10–20 kDa (Nadir dialysis membrane; Carl Roth, Karlsruhe, Germany). The reaction product was dialyzed against 10 L of 1 mM HCl, twice against 10 L of 1 mM HCl containing 1% NaCl, and again twice against 10 L of 1 mM HCl. The dialysis product was frozen at −80 °C and lyophilized (Gamma 1-16 LSC, Christ, Osterode, Germany). To ensure complete removal of uncoupled l-cysteine, a sample omitting EDAC and NHS was prepared as control following the same procedure.

#### 2.2.2 Synthesis of CS-Cys-MNA

S-protection with 6-MNA was achieved via a thiol/disulfide-exchange reaction according to a slightly modified protocol previously applied by Laffleur et al.^17,21^ Briefly, 200 mg of CS-Cys was dissolved in 50 mL of a DMF/water mixture in a ratio of 7:3 and adjusted to pH 6.2 by the addition of 1 M HCl. After dissolving 50 mg of 6,6′-DTNA in 50 mL of DMF, the solution was added dropwise to the solution of CS-Cys. The mixture was stirred for 5 h at room temperature while maintaining the pH between 6.0 and 6.2 using 1 M NaOH. The product was transferred to a dialysis tube with a molecular weight cut-off of 10–20 kDa and dialyzed three times against a mixture of 3 L of demineralized water containing 1% NaCl and 0.5 L of DMSO. Subsequently, salt and DMSO were removed by dialyzing five times against 10 L of demineralized water. The final product was obtained after lyophilization.

#### 2.2.3 Synthesis of CS-Cys-Cys

To substitute 6-MNA with l-cysteine, 100 mg of l-cysteine was dissolved in 25 mL of demineralized water. The l-cysteine solution was added dropwise after the previous reaction between CS-Cys and 6,6′-DTNA was completed. The pH was maintained between 6.0 and 6.2 using 1 M NaOH and the mixture was stirred for a further 90 min. The product was dialyzed in the same way as CS-Cys-MNA. The final product was obtained after lyophilization.

#### 2.2.4 Characterization of Synthesized Compounds

##### 2.2.4.1 Nuclear Magnetic Resonance Spectroscopy (NMR)

All ^1^H NMR measurements were performed on a “Mars” 400 MHz Avance 4 Neo spectrometer from Bruker Corporation (Billerica, MA, 400 MHz) in D_2_O with the addition of 1% acetic acid-d_4_.

##### 2.2.4.2 Fourier-Transform Infrared Spectroscopy (FT-IR)

FT-IR spectra were recorded on a Spectrum Two spectrometer (PerkinElmer, Beaconsfield, U.K.) using four scans at a resolution of 1 cm^–1^ recorded from 4000 to 400 cm^–1^. The depicted spectra are the mean of applied scans.

##### 2.2.4.3 Ellman’s Test

Ellman’s test was used to determine the degree of thiolation.^22^ Briefly, 0.5–1.0 mg of each polymer were hydrated in 250 μL of demineralized water. After dilution with 250 μL of 0.5 M phosphate buffer, pH 8.0, 500 μL of Ellman’s reagent containing 3 mg of DTNB in 10 mL of 0.5 M phosphate buffer, pH 8.0, were added. After 90 min of incubation, protected from light and at room temperature, the samples were centrifuged for 5 min at 13 400 rpm. Aliquots of 100 μL were transferred to a UV plate, and absorbance was measured at 450 nm using a Tecan Spark (Tecan, Grödig, Austria). Calibration curves were established using l-cysteine-HCl × H_2_O (*R*^2^ ≥ 0.99).

To quantify disulfide bonds, samples were hydrated in 350 μL of demineralized water and subsequently diluted with 150 μL of 0.5 M Tris buffer, pH 7.6. Disulfide bonds were reduced with an excess of sodium borohydride being dissolved in demineralized water at a concentration of 40 mg/mL. After discarding the unreacted sodium borohydride by adding 5 M HCl, the solution was neutralized using a 1 M phosphate buffer, pH 8.0. Ellman’s test was then conducted as described above.

##### 2.2.4.4 MNA Test

To prove, the removal of uncoupled 6-MNA and quantify the 6-MNA immobilized on thiolated polymers, a test previously described by Lupo et al.^22^ was applied. First, 0.5–1.0 mg of each S-protected thiolated polymer was hydrated in 250 μL of demineralized water. After dilution with 250 μL of 0.5 M phosphate buffer, pH 8.0, 500 μL of a freshly prepared 0.2% (m/v) L-glutathione solution was added and incubated for 90 min protected from light at room temperature. To quantify the unbound 6-MNA, samples in the absence of L-glutathione were prepared in the same way. Aliquots of 100 μL were transferred to a UV plate and measured at 354 nm. Calibration curves were established using 6-MNA (*R*^2^ ≥ 0.99).

#### 2.2.5 Preparation of Nanoparticles

SLN were prepared using an emulsification ultrasonication method.^23,24^ Briefly, 500 mg of cetyl palmitate and 50 mg of egg lecithin were molten at 65 °C. To prepare labeled SLN, 5 mg of LGR was added to the lipid phase. As an aqueous phase, 200 mg of Pluronic F127 was dissolved in 10 mL of demineralized water. When applying coatings, 40 mg of polymeric coating material was also added to the aqueous phase. The aqueous phase was heated to 65 °C and added to the lipid blend. The mixture was pre-emulsified for 30 s by means of high shear homogenization at 27 000 rpm (IKA EuroTurrax T206, Staufen, Germany). The formed pre-emulsion was sonicated twice for 60 s, applying an amplitude of 80% with a Hielscher UP200H (Hielscher, Teltow, Germany). The resulting hot nanoemulsion was immediately transferred to an ice bath. After cooling, SLN were used without further purification.

When applying CS coating, 1% acetic acid (m/v) served as an aqueous phase. When applying coatings of CS-Cys and CS-Cys-Cys, demineralized water was used and in the case of CS-Cys-MNA, 0.5 M phosphate buffer was added with the pH adjusted to 8.0. SLN were subsequently prepared as described above.

#### 2.2.6 Particle Characterization

##### 2.2.6.1 Particle Size, Polydispersity Index (PDI), and ζ Potential

The hydrodynamic diameter of SLN formulations expressed as Z-average and PDI were derived from the autocorrelation fit of the data obtained from dynamic light scattering (DLS) using the cumulant method. Therefore, SLN dispersions were diluted 1:100 in 10 mM PBS, pH 7.4, and transferred to disposable polystyrene cuvettes. Samples were measured using a He–Ne laser with a wavelength of 633 nm and a backscattering angle of 173°. ζ potential was measured via electrophoretic light scattering after diluting SLN dispersions 1:500 in demineralized water by applying the Smoluchowski relation. Samples were measured at a scattering angle of 12.8° with the aid of a dip cell (Malvern Instruments, Malvern, U.K.). Each replicate of both methods consisted of three consecutive runs and was carried out at 37 °C using a ZetaSizer Nano ZSP (Malvern Instruments, Worcestershire, U.K.). To determine storage stability, SLN formulations were measured following the same protocol after storage at 4 °C for 30, 90, and 180 days.

##### 2.2.6.2 Shape and Surface Morphology

The shape and surface morphology were investigated using an energy filter transmission electron microscopy (EFTEM). Therefore, SLN dispersions were mounted on 200 mesh, Formvar/carbon-coated copper grids (Balzers Union, Liechtenstein), dried, and examined with a Zeiss Libra 120 (Carl Zeiss AG, Oberkochen, Germany). Images were obtained with a 2 x 2k high-speed camera (Troendle, Germany) and ImageSP software (Troendle, Germany). SLN formulations were diluted 1:10 with demineralized water before measurements.

##### 2.2.6.3 Powder X-ray Diffraction Analysis (PXRD)

Undiluted SLN dispersions and single components of each dispersion as bulk material were analyzed using PXRD. The samples were measured on a *Mylar* (6 μ) foil. The PXRD patterns were obtained using an X’Pert PRO diffractometer (PANalytical, Almelo, the Netherlands) equipped with a θ/θ coupled goniometer in transmission geometry, a Cu Kα_1,2_ radiation source with a focusing 0.5° divergence slit and 0.02° Soller slit collimator on the incident beam side, a 2 mm antiscattering slit and 0.02° Soller slit collimator on the diffracted beam side mirror, and a solid-state PIXcel detector. The patterns were recorded at a tube voltage of 40 kV and a tube current of 40 mA, with a step size of 2θ = 0.013° with 80 s (components) or 400 s (SLN formulations) per step in the 2θ range between 2 and 40°.

#### 2.2.7 Mucus Collection and Purification

Mucus was scraped off from the freshly excised porcine intestine, which was obtained from a local slaughterhouse. Intestinal segments that contained food residues, as well as mucus that appeared yellow, were discarded. The crude mucus was frozen at −20 °C until purification. To purify the collected mucus, the crude mucus was diluted 1–5 with 0.1 M NaCl solution and gently stirred for 1 h at 10 °C. After centrifugation at 10 400*g* and 4 °C (Sigma 3-18KS, Sigma Laborzentrifugen, Osterode am Harz, Germany) for 2 h, the supernatant and granular material on the bottom were discarded. Subsequently, the mucus was diluted with half of the volume of 0.1 M NaCl. Stirring and centrifugation were repeated as described above. The supernatant was removed and the purified mucus was stored at −20 °C until further use.

For the mean of single-particle tracking (SPT), an altered protocol was applied as described by Le-Vinh et al.^25^ In brief, the fresh porcine small intestine was put on ice immediately after being collected. Then, it was rinsed with ice-cold 67 mM phosphate buffer, pH 6.7, containing 0.02% w/v sodium azide and a mix of protease inhibitors to remove the debris. The mucus was collected by gently scraping the epithelial surface of the jejunal segment of the intestine with a plastic scraper, collected in aliquots, and directly put on ice. The debris was further removed by extracting the mucus overnight at 16 °C under gentle stirring in 7 volumes of extraction buffer adjusted to pH 6.5 containing 10 mM sodium phosphate, 4 M guanidinium hydrochloride, 5 mM EDTA, 5 mM *N*-ethylmaleimide, and 0.02% (w/v) sodium azide. The precipitated material was collected by centrifugation for 30 min at 22 104*g* and 10 °C and re-extracted in the same manner with 10 volumes of the extraction buffer. After another centrifugation, the insoluble precipitate was collected and stored at −80 °C prior to use.

#### 2.2.8 Stability in Biorelevant Media

The stability of SLN formulations was investigated in simulated intestinal fluid (SIF), simulated gastric fluid (SGF), fasted-state simulated intestinal fluid (FaSSIF), and fed-state simulated intestinal fluid (FeSSIF). The SIF and SGF were prepared according to USP specifications. FaSSIF and FeSSIF were prepared according to the supplier’s manual. To determine stability, SLN were diluted 1:100 in each medium. After 4 h of incubation at 37 °C, SLN were analyzed via DLS as described previously.

To determine changes in the characteristics of SLN getting into contact with mucus, they were incubated with a dilution of purified mucus. Briefly, 100 mg of purified mucus was diluted in 1 mL of 10 mM PBS, pH 7.4. Equal volumes of SLN dispersions and mucus dilution were incubated at 37 °C for 4 h. The mixtures were diluted 1:50 with 10 mM PBS, pH 7.4, before measuring via DLS.

#### 2.2.9 Rheological Measurements

To further investigate the interaction of mucus and SLN formulations, rheological measurements were conducted. Therefore, 500 μL of purified mucus and 500 μL of undiluted SLN dispersion were gently mixed using a spatula. After 4 h of incubation at 37 °C, samples were transferred to a Haake Mars plate-plate rheometer (Thermo Scientific, Vienna, Austria). Strain sweep measurements were conducted at a frequency of 1 Hz, whereas frequency sweep measurements were conducted at a shear rate of 0.1 Pa.

#### 2.2.10 Single-Particle Tracking

To evaluate the diffusion characteristics of SLN in purified porcine intestinal mucus, SPT was employed. LGR-labeled SLN were diluted 1:500 with 10 mM PBS, pH 7.4, to yield a lipid content of 0.01% (w/v). Subsequently, SLN dilution was added to 30 μL of purified mucus in an amount that would yield a final concentration of 3% (mix 1) or 20% (v/v) (mix 2), respectively. Subsequently, the mixture was gently stirred using a pipette and equilibrated for 30 min at room temperature. To perform SPT experiments, 5 μL of the mucus–SLN mixture was inserted into a custom-made imaging chamber and placed onto the microscope stage. The sample was left on the microscope stage for a further 5 min so that the mucus–SLN mixture in the imaging chamber could reach equilibrium from the motion of handling. SLN diffusion was measured by tracking the positions of the labeled SLN using an sCMOS camera(Hamamatsu digital camera C11440, ORCA-ash 4.0, Japan) mounted on an inverted wide-field microscope (Dmi8, Leica, Germany) with a 63Ö/1.2NA objective, appropriate filters, and with an attached Lumencor Spectra × fluorescence illumination system (Olympus). For each sample, 90 image sequences were acquired with LASX software (Leica) at a temporal resolution of 10 ms to obtain at least 100 frames of particle trajectories.

To obtain particle trajectories, the image sequences were analyzed using the feature point detection and tracking algorithm of the ParticleTracker ImageJ plugin^26^ and the ImageJ-Matlab extension.^27^ Trajectories longer than 30 frames were analyzed using a custom-written Matlab program. A minimum of 400 trajectories was assessed for each formulation. The coordinates of SLN centroids were used to determine the time-averaged mean-squared displacement (MSD).
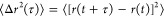
1Particle position at time *t* is referred to as *r*(*t*), whereas τ is the time lag. From the ensemble-averaged MSD, the generalized time-independent diffusion coefficient and the dimensionless anomalous exponent were determined via a linear fit of the log transformation of eq 2.(28)
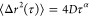
2Anomalous diffusion can be efficiently estimated when an ensemble of trajectories is available.^29^ The particle mobility was further evaluated via calculation of the effective diffusion coefficient *D*_e_ for a time scale of τ = 0.5 s.
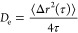
3*D*_e_ was normalized to the mean diffusion coefficient *D*_w_ measured in demineralized water. *D*_w_ was determined via a weighted linear fit to the ensemble-averaged MSD. Particles were considered immobile when ⟨Δ*r*^2^(τ_0.5 *s*_)⟩ was less than 13 nm, which is below the tracking resolution at a time lag of 0.5 s. Particles displaying an MSD below their diameter at that time scale were ranked as hindered, and particles were considered diffusive when *D*_e_/*D*_w_ was approximately 1.^28^

#### 2.2.11 Mucus Diffusion via Rotating Tube Assay

Mucus diffusion assay was conducted as previously described by Akkus et al.^30^ with slight modifications. Briefly, silicon tubes with an inner diameter of 30 mm were cut into pieces of 5 mm in length. Tubes were filled with 150 μL of purified mucus and closed with a silicone plug on one end. Subsequently, 50 μL of labeled SLN dispersion was deposited on top of the mucus. The tube was closed on the other end and subjected to horizontal rotation with 50 rpm (IKA RM 18, IKA, Staufen, Germany). After 24 h of rotation at 37 °C, the tubes were frozen at −80 °C. Frozen tubes were cut into slices of 2 mm in length. Each slice was placed in 500 μL of DMF to extract LGR. The tube and undissolved parts were separated from the dye solution by means of centrifugation for 5 min at 12 000*g*. LGR was measured via fluorescence at λ_ex_ = 570 nm and λ_em_ = 610 nm.

#### 2.2.12 Mucosal Retention Assay

The porcine intestinal mucosa was lengthwise opened and cut into pieces of 2 × 5 cm^2^. Each piece was glued on a half-cut 50 mL falcon tube. After depositing 200 μL of labeled SLN dispersion on the mucosa, they were incubated horizontally for 10 min. Subsequently, each mucosa was mounted at an angle of 45° and rinsed with 10 mM PBS, pH 7.4 using a flow of 1 mL/min at 100% relative humidity. At predetermined time points, the amount of LGR that was washed off was measured. Therefore, the collected PBS was diluted 1:5 in DMF to extract LGR. After 120 min, LGR that remained on the mucosa was extracted by washing the mucosa with 10 mL DMF. All collected samples were measured via fluorescence at λ_em_ = 570 nm and λ_ex_ = 610 nm.

#### 2.2.13 Statistical Analyses

When two sets of data were compared with each other, Student’s *t*-test was applied. For the comparison of more than two data sets, one-way analysis of variance (ANOVA) and Bonferroni post hoc test were applied. GraphPad Prism 5 software was used for all statistical analyses.

## 3 Results

### 3.1 Synthesis and Characterization of Thiolated CS

**Figure 1:**
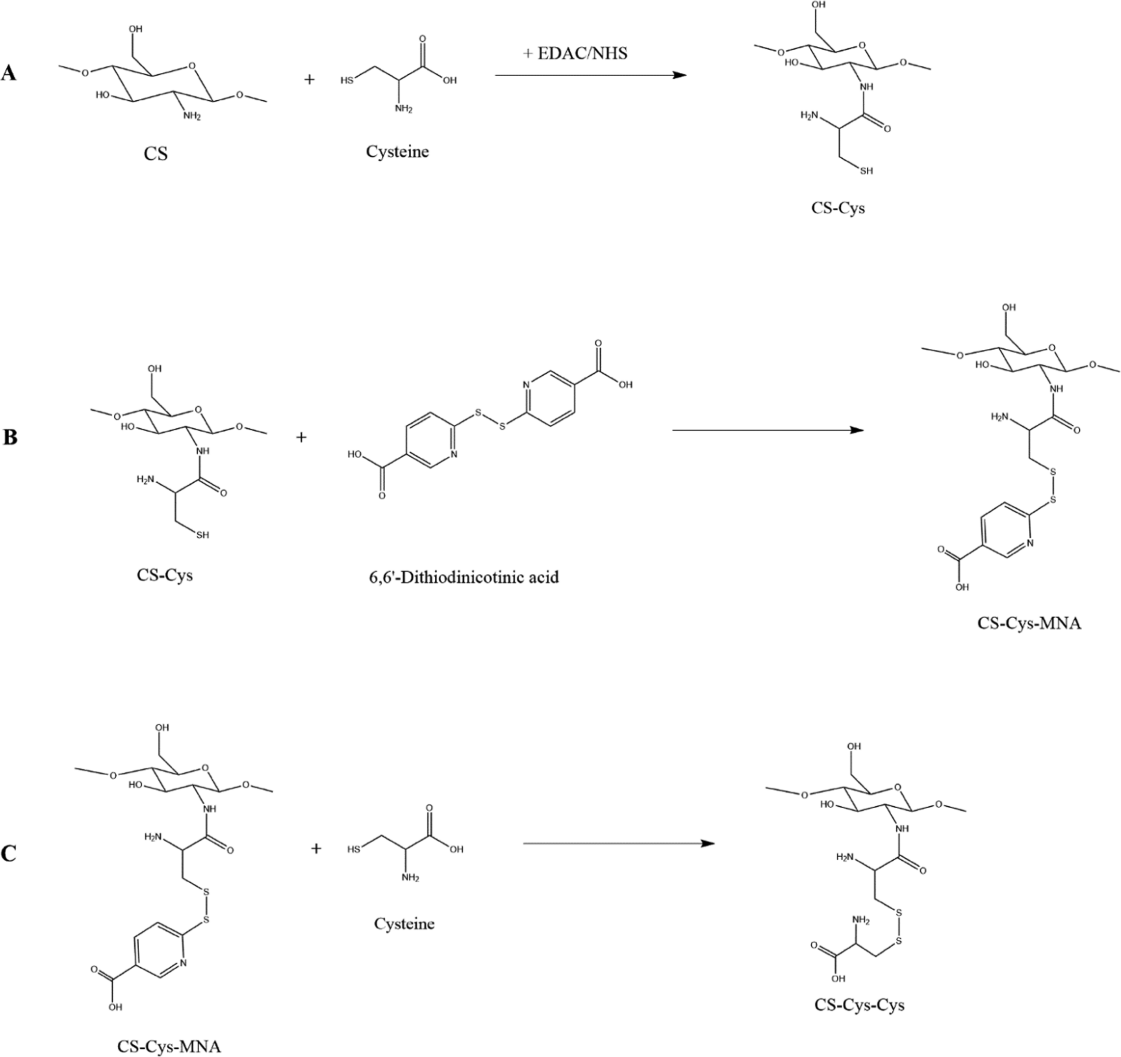
Synthesis pathways for CS-Cys (A), CS-Cys-MNA (B), and CS-Cys-Cys (C).

CS-Cys was prepared via amide bond formation mediated by EDAC × HCl and NHS. Both S-protected thiolated polymers were subsequently prepared using a thiol/disulfide-exchange reaction. The synthesis pathways are provided in Figure 1. The progress of both thiol/disulfide reactions is indicated by yellow color due to the release of 6-MNA. The structure of the produced CS derivatives was studied by ^1^H NMR (Figure S1) and FT-IR spectroscopy (Figure S2). Compared to the NMR spectrum of native CS, CS-Cys shows a peak at approximately 3.0 ppm due to the methylene protons next to the thiol group. This peak is slightly shifted downfield due to the adjacent disulfide bond formation when recording S-protected CS derivatives. The peaks between 7.5 and 8.2 ppm are clearly indicating the presence of the aromatic protons of nicotinic acid in CS-Cys-MNA. The disappearance of the aromatic signals from the spectrum of CS-Cys-Cys and the increased intensity of the methylene peak next to the disulfide bond confirm the formation of the oxidized cysteine dimer. All FT-IR spectra show distinct bands at 1640, 1530, 1380, and 1310 cm^–1^. As all polymers are still acetylated, they exhibit amide bonds to which these bands can be related. However, in the case of thiolated compounds, a distinct increase in intensity was found for the peak at 1530 cm^–1^ due to structural changes. Further, the number of free thiol groups, disulfide bonds, and 6-MNA immobilized on thiolated CS was quantified via colorimetric assays (Table 1). Ellman’s test revealed a significantly higher number of free thiol groups and a significantly smaller number of disulfide bonds on CS-Cys than in both S-protected thiolated CS. A control reaction was conducted with CS and l-cysteine but omitting NHS and EDAC × HCl did not lead to any detectable thiol groups. Thus, a quantitative removal of free l-cysteine can be assumed. CS-Cys-MNA exhibited a significant discrepancy between the number of disulfide bonds and the bound 6-MNA, whereas no detectable 6-MNA was found in CS-Cys-Cys.

**Table 1:** Amount of Free Thiol Groups, Disulfide Bonds, and Bound MNA (*n* ≥ 3)^a^

	amount of free thiol groups ± SD [μmol/g polymer]	amount of disulfide bonds ± SD [μmol/g polymer]	amount of bound MNA ± SD [μmol/g polymer]
CS-Cys	590.6 ± 72.4*	256.4 ± 67.4*	not tested
CS-Cys-MNA	38.6 ± 4.7	513.6 ± 90.4	195.8 ± 98.3
CS-Cys-Cys	82.8 ± 24.5	662.2 ± 146.7	below detection range

a Significant differences to other thiolated polymers are indicated with *p ≤ 0.05.

### 3.2 Preparation, Characteristics, and Stability of SLN

**Figure 2:**
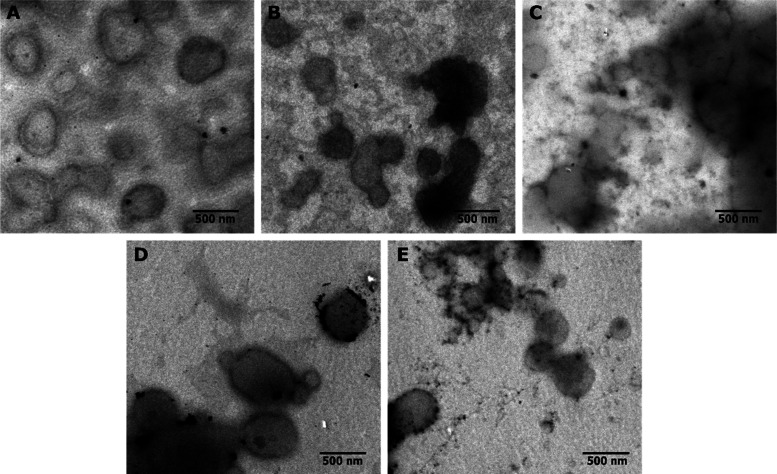
EFTEM images of plain (A), CS-coated (B), CS-Cys-coated (C), CS-Cys-MNA-coated (D), and CS-Cys-Cys-coated SLN (E).

The spherical morphology of SLN was confirmed by EFTEM (Figure 2). Plain and CS-coated SLN were noticeably more homogeneous than SLN coated with thiolated polymers. Particle sizes observed via EFTEM were in good agreement with those measured by DLS. DLS revealed hydrodynamic sizes below 600 nm for all formulations, as well as monomodal size distributions (Table 2). None of the formulations underwent significant changes in size or PDI over the course of 4 h. Further, different coating materials led to different ζ potential values.

**Table 2:** Hydrodynamic Size, PDI, and ζ Potential of SLN Formulations (*n* ≥ 3)

	size ± SD [nm]	PDI ± SD	ζ potential ± SD [mV]
plain	244.7 ± 30.7	0.24 ± 0.05	–37.8 ± 4.7
CS	261.6 ± 7.4	0.20 ± 0.02	54.7 ± 5.6
CS-Cys	402.3 ± 46.2	0.27 ± 0.02	37.3 ± 5.1
CS-Cys-MNA	533.9 ± 167.7	0.34 ± 0.05	–10.7 ± 6.7
CS-Cys-Cys	422.0 ± 31.2	0.32 ± 0.06	27.3 ± 9.2

Cetyl palmitate exhibited sharp diffraction reflections, unambiguously indicating that the lipid is present in the crystalline state. All of the PXRD patterns recorded for the SLN formulations showed the characteristic cetyl palmitate reflection positions (Figure S3). Thus, after formulating the lipid in the form of SLN, no change in the crystalline solid-state form was observed. Furthermore, Pluronic F127 and egg lecithin showed some crystalline features, whereas coating materials only exhibited characteristic features for amorphous compounds.

**Figure 3:**
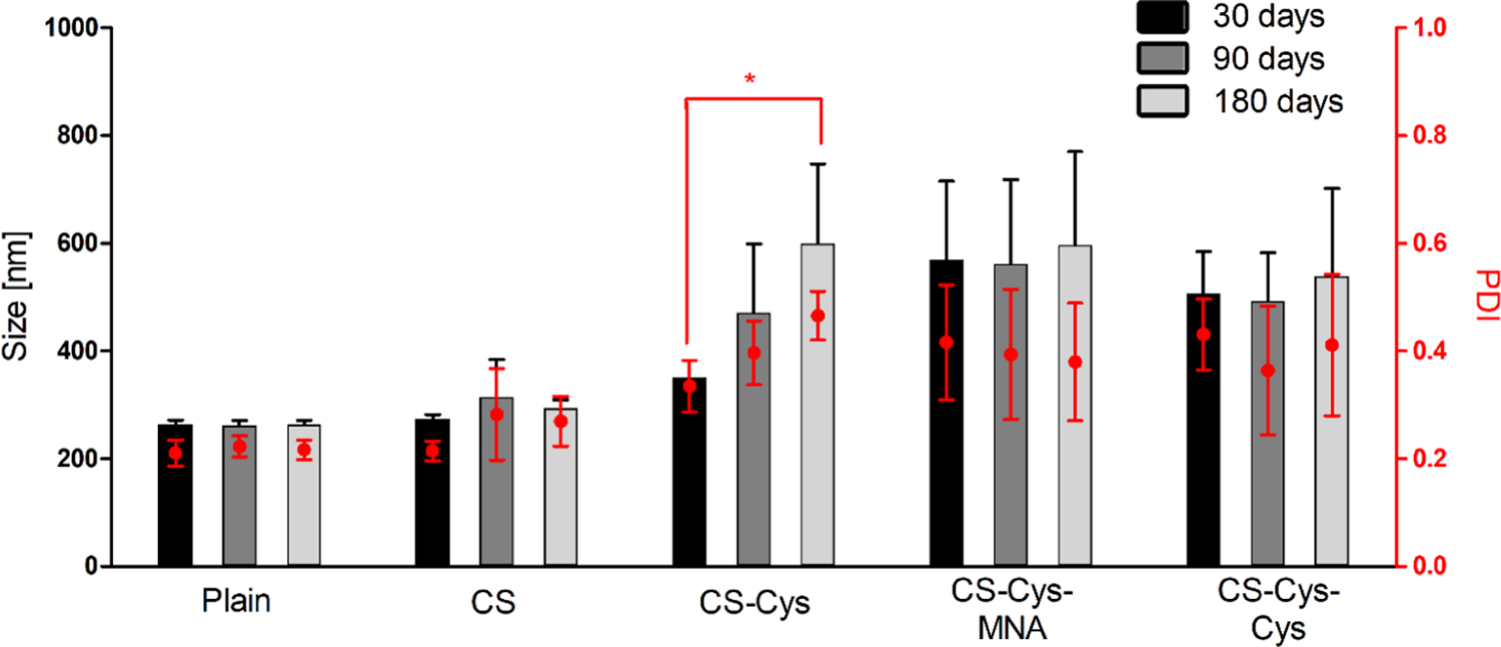
Hydrodynamic size and PDI of SLN formulations after 30, 90, and 180 days of storage at 4 °C (*n* ≥ 3). *p ≤ 0.05 indicates differ significantly.

After storage of only a few hours, phase separation of CS-Cys-MNA-coated SLN was observed. However, particles showed stable characteristics when being re-dispersed before measurement. All other formulations were visibly stable over 180 days. Still, the size and PDI of CS-Cys-coated SLN were continuously increasing over time (Figure 3).

**Figure 4:**
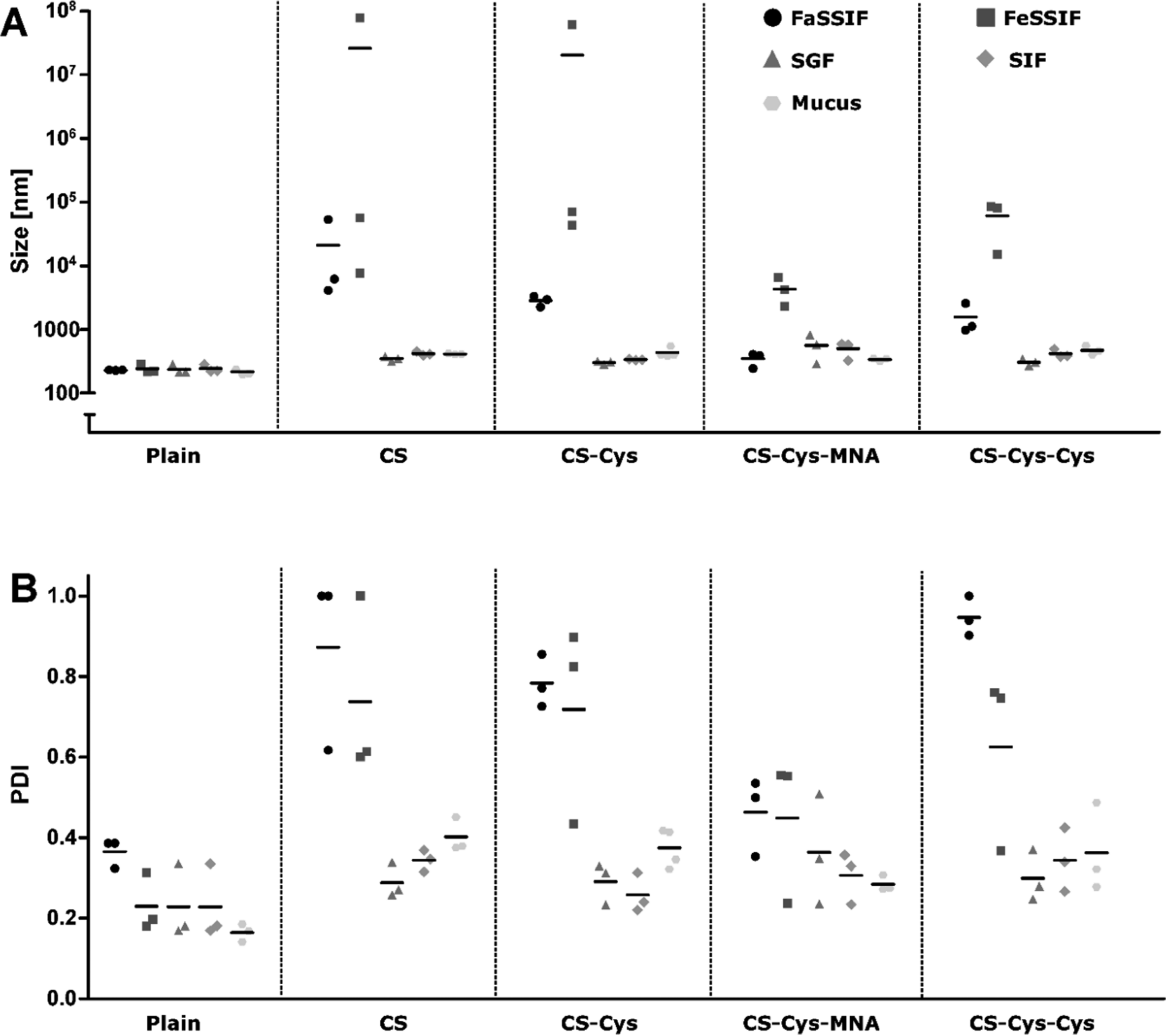
Hydrodynamic size (A) and PDI (B) of SLN formulations in FaSSIF, FeSSIF, SGF, SIF, and mucus after 4 h of incubation at 37 °C (*n* ≥ 3).

All SLN formulations were stable in the enzyme-containing media SGF and SIF, and in diluted mucus (Figure 4). CS-, CS-Cys- and CS-Cys-Cys-coated SLN showed a significant increase in size and PDI in FaSSIF. This increase was even more pronounced in FeSSIF. CS-Cys-MNA-coated SLN showed an increase in size in FeSSIF but not in FaSSIF, whereas PDI increased in both media.

### 3.3 Rheological Measurements

**Figure 5:**
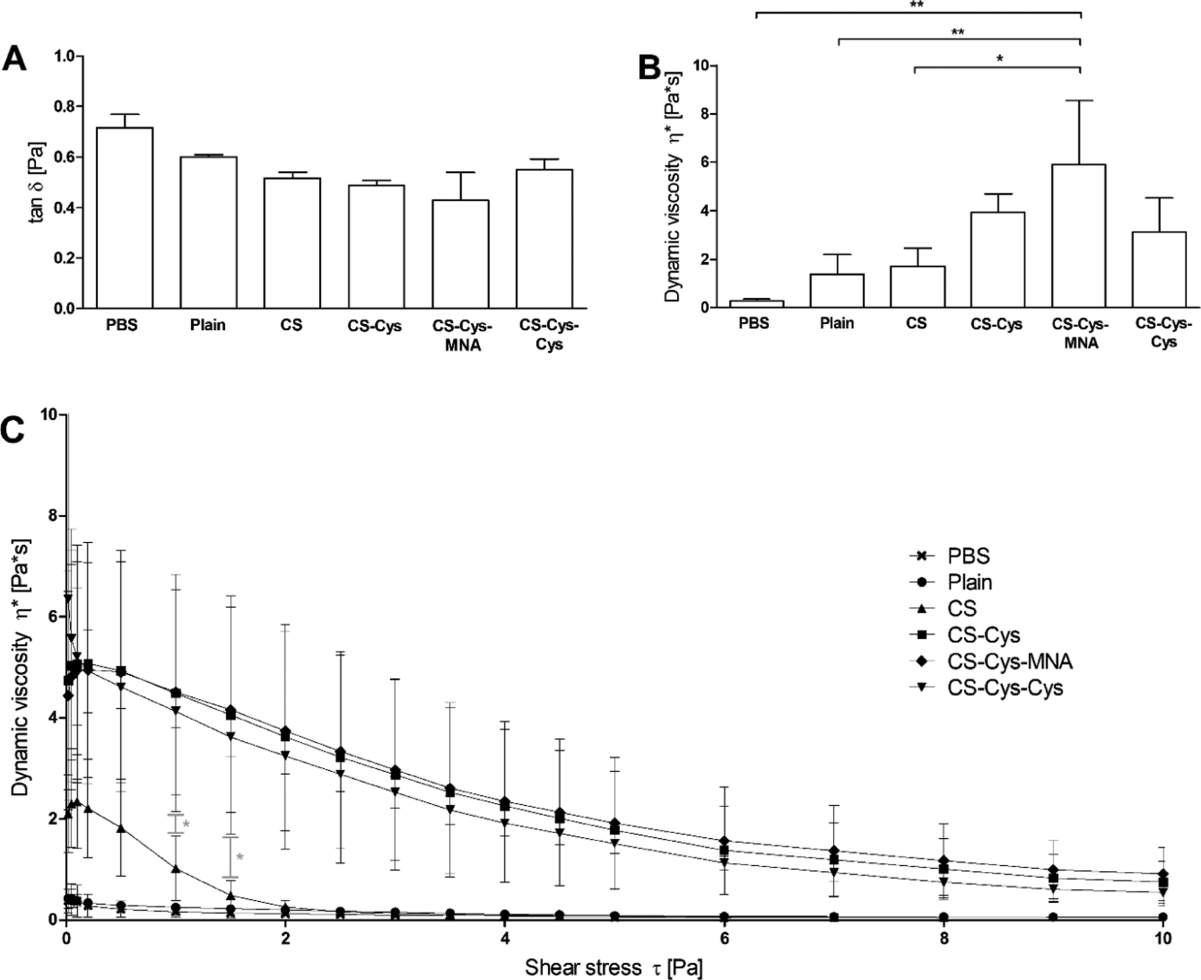
Loss tangent tan δ (A) and dynamic viscosity η* (B) measured at a frequency of 1 Hz and a shear rate of 0.1 Pa and dynamic viscosity η* as a function of shear rate τ measured at a frequency of 1 Hz (C). All measurements were conducted after 4 h of incubation at 37 °C (*n* ≥ 3). Significant differences: *p ≤ 0.05 and **p ≤ 0.01.

The results obtained by rheological measurements are shown in Figure 5. The loss tangent tan δ and dynamic viscosity η* were in good agreement as they showed an inverse correlation. Only CS-Cys-MNA-coated SLN led to a significantly increased viscosity in comparison to plain and CS-coated SLN. When applying strain sweep measurements, all SLN coated with thiolated CS maintained a substantially higher dynamic viscosity with increasing shear forces than the CS-coated and plain SLN.

### 3.4 Single-Particle Tracking

**Figure 6:**
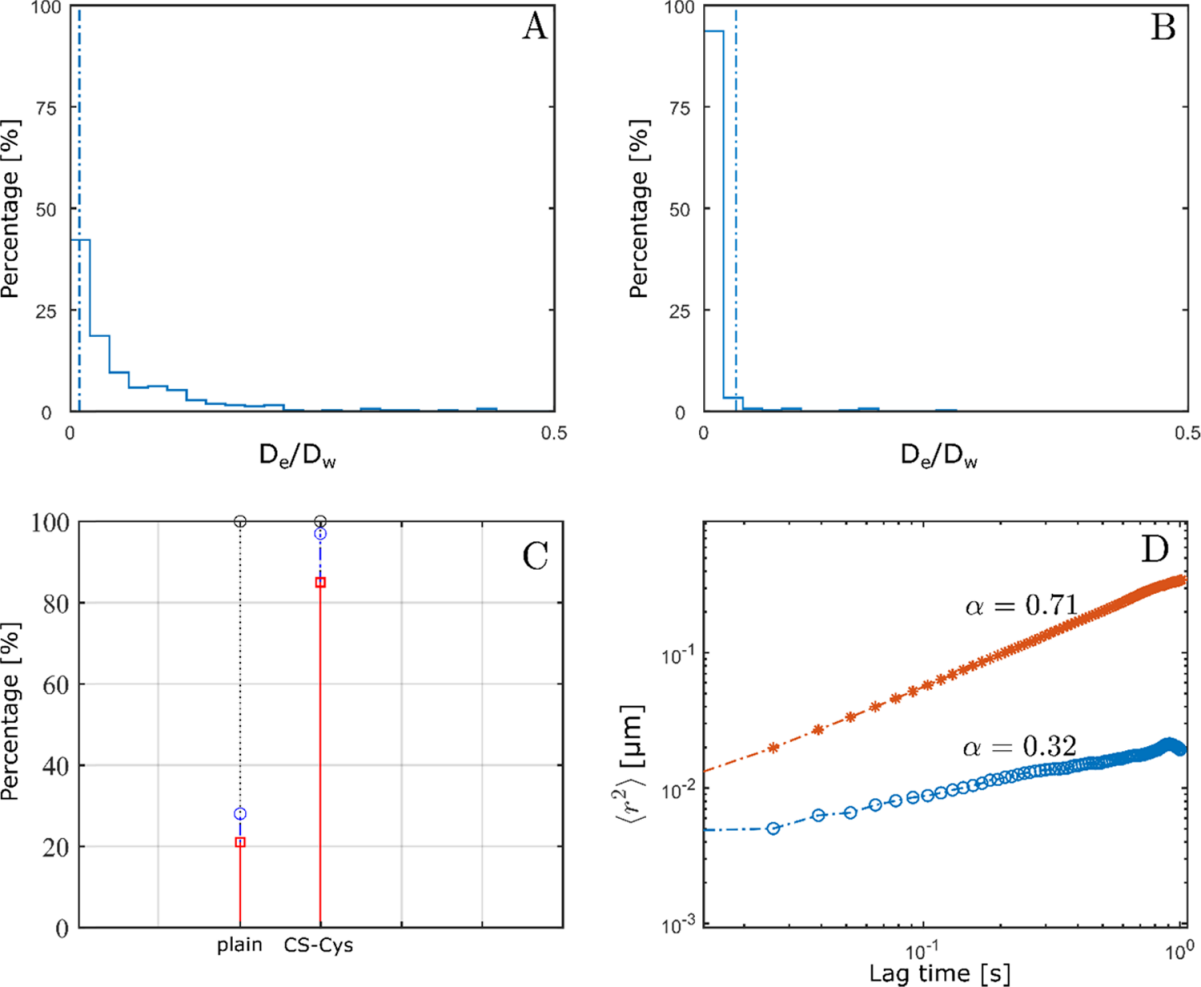
Distribution of normalized effective diffusion coefficients of plain (A) and CS-Cys-coated SLN (B) in mix 1. Particles behind the dashed line are considered as immobile. Distribution of particles being immobilized (red line), hindered (blue line), and diffusive (gray line) (C) and log–log plot of MSD and lag time of plain (red line) and CS-Cys-coated SLN (blue line) as well as corresponding anomalous coefficients indicating the type of motion (D).

Mucus permeating properties of SLN formulations were investigated as a function of two different SLN/mucus ratios (mix 1, mix 2). Mix 1, a dilution typically used in SPT experiments,^31^ allowed distinction into two groups. On the one hand, plain and CS-Cys-MNA-coated SLN having diffusion coefficients of 1.0 × 10^–1^ and 0.5 × 10^–1^ μm/s, respectively, and on the other hand, CS-coated, CS-Cys-coated, and CS-Cys-Cys-coated SLN having up to 23-fold lower mean diffusion coefficients of 4.4 × 10^–3^ μm/s than the plain SLN. In Figure 6, CS-Cys-coated SLN are shown as a representative formulation for the ones exhibiting hindered diffusion as evident by the decreased anomalous exponent. However, the diffusion coefficients and percentages of immobilized particles for these formulations differed less than 15% in mix 1. Hence, to excavate differences, a higher dilution of mucus (mix 2) was applied. A higher dilution might promote swelling of the mucus hydrogel and thereby reduce the effect of size-dependent impediment of particle diffusion. Thus, adhesive forces between mucus and particles would become the dominant influence on particle diffusion behavior.

**Figure 7:**
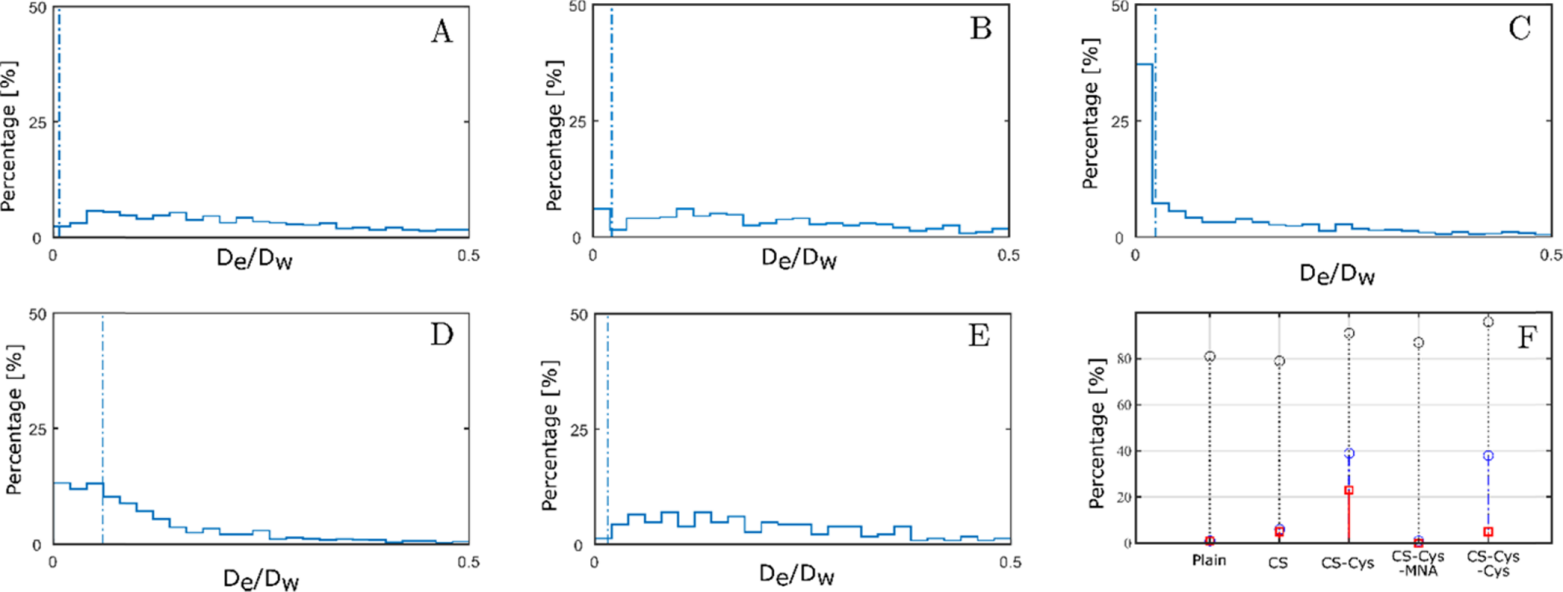
Distribution of normalized effective diffusion coefficients of plain (A), CS-coated (B), CS-Cys-coated (C), CS-Cys-MNA-coated (D), and CS-Cys-Cys-coated SLN (E) in mix 2. Particles behind the dashed line are considered as immobile. Distribution of particles in each formulation being immobilized (red line), hindered (blue line), and diffusive (gray line) (F).

Indeed, particles diffusing in mix 2 exhibited larger differences with respect to their percentage of immobilized particles (Figure 7). CS-Cys-coated and CS-Cys-Cys-coated SLN displayed the largest percentage of strongly hindered particles. Approximately 40% of these particles had normalized effective diffusion coefficients corresponding to an MSD less than the particle diameter.^32^ Moreover, the percentage of immobilized CS-Cys-coated SLN was more than 4-fold larger than in the case of CS-Cys-Cys-coated SLN. CS-coated SLN had similar percentages of immobilized particles as CS-Cys-Cys-coated SLN but a lower percentage of hindered particles. Anomalous exponents of all formulations in mix 2 were less than 1 indicating subdiffusive diffusion characteristics despite the higher dilution of mucus.

### 3.5 Mucus Diffusion Via Rotating Tube Assay

**Figure 8:**
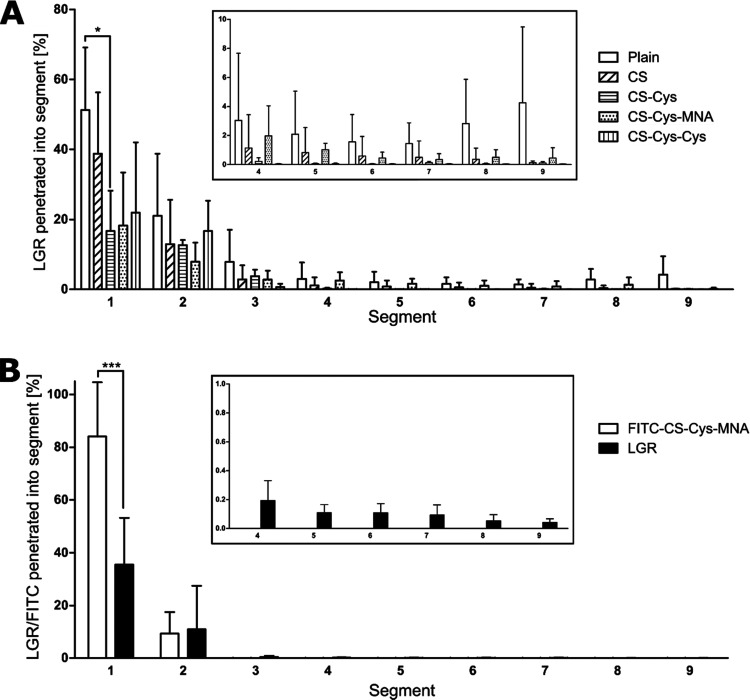
Mucus diffusion of plain, CS-coated, CS-Cys-coated, CS-Cys-MNA-coated, and CS-Cys-Cys-coated SLN (A) and of FITC-CS-Cys-MNA-coated SLN (B) after 24 h of incubation in a rotating tube assay at 37 °C (*n* ≥ 3). Significant differences: *p ≤ 0.05 and *** *p* ≤ 0.001.

In accordance with SPT, plain, CS- and CS-Cys-MNA-coated SLN could diffuse into rear segments of the mucus layer while nearly no CS-Cys- and CS-Cys-Cys-coated SLN could reach into segment 5 and posterior of it (Figure 8). To further investigate the beneficial diffusion behavior of CS-Cys-MNA-coated SLN, CS-Cys-MNA was labeled with FITC as conducted by Knoll et al.^33^ and the experiment was carried out in the same way. Thus, diffusion of CS-Cys-MNA and SLN could be distinguished from each other. While a significantly higher amount of CS-Cys-MNA was present in the front segment, from segment 3 onwards, only LGR-labeled SLN were recovered.

### 3.6 Mucosal Retention Assay

**Figure 9:**
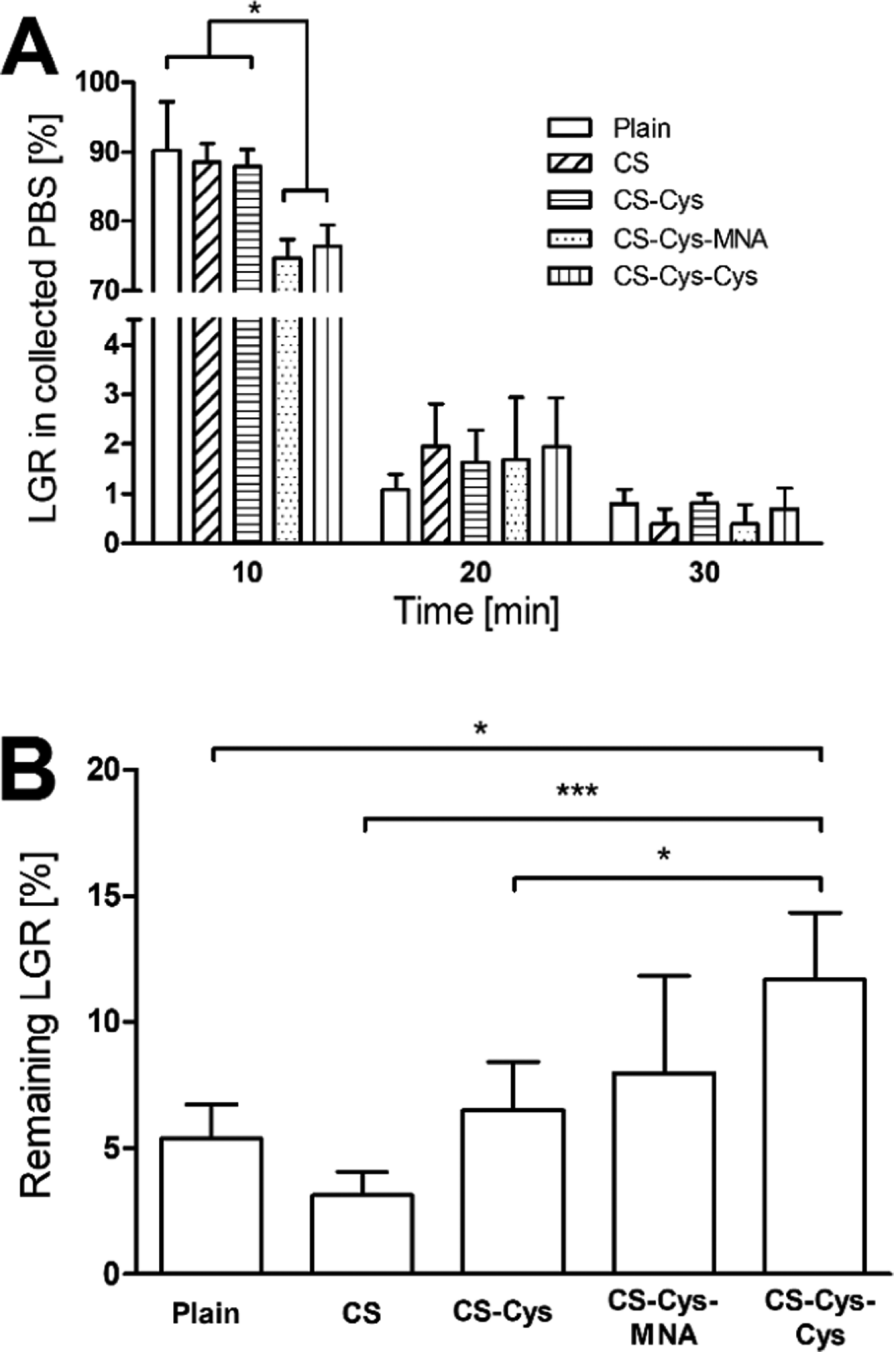
LGR collected in PBS (A) and LGR remaining on porcine intestinal mucosa after 120 min (B) in mucosal retention assay (*n* ≥ 3). Significant differences: * *p* < 0.05 and ***p < 0.001.

The results obtained via the mucosal retention assay are shown in Figure 9. Significantly lower amounts of LGR are washed off during the first 10 min when CS-Cys-MNA- and CS-Cys-Cys are applied as coating materials. Consequently, these coatings enable a higher LGR amount to be retained on the mucosa.

## 4 Discussion

### 4.1 Synthesis of Thiolated CS

In recent studies, a disulfide bond-bearing ligand was bound to a polymer backbone^19,20^ to yield less-reactive S-protected thiolated polymers, whereas a thiol/disulfide-exchange reaction was used in this study. The latter was not applied for less-reactive S-protected thiolated polymers, yet it is common for 6-MNA-S-protection.^17,34^ FT-IR spectra are in line with previously synthesized S-protected thiolated CS derivatives modified with *N*-acetylcysteine.^20^ Moreover, successful immobilization of l-cysteine on CS as well as subsequent protection of the introduced free thiol group with 6-MNA and l-cysteine, respectively, was confirmed via ^1^H NMR and colorimetric assays. Thus, the exchange of 6-MNA by adding l-cysteine proved to be a suitable way to obtain less-reactive S-protected thiolated polymers. However, the amount of bound 6-MNA was lower than in studies using a disulfide-bearing ligand.^20,22^ Moreover, an entire S-protection could not be achieved. Nonetheless, the amount of free thiol groups, as well as of disulfide bonds, in the case of CS-Cys and CS-Cys-MNA is in good agreement with values found in the literature using similar synthesis protocols.^9,17,35^ The discrepancy between the bound MNA and the number of disulfide bonds can be explained by a cross-linking of the polymer via disulfide bonds during synthesis or purification of the product.

### 4.2 Preparation, Characteristics, and Stability of SLN

All formulations showed the ability to form nanocarriers as proven by DLS and EFTEM. Moreover, the solid state and the maintained crystallinity of the solid matrix were confirmed via PXRD. Further, distinct changes in ζ potential when introducing CS and its derivatives prove successful coating.^7^

Decreased storage stability found for CS-Cys-coated SLN is likely related to oxidation of free thiol groups.^16^ Since the absolute ζ potential values are comparatively low, limited electrostatic repulsion results in the flocculation of CS-Cys-MNA-coated SLN. In addition, increased hydrophobicity of the coating materials could contribute to phase separation.

None of the enzyme-containing compendial media had noticeable effects on SLN formulations, whereas FaSSIF and FeSSIF led to a tremendous increase in size and PDI of the positively charged SLN. Aggregation of these formulations can likely be attributed to charge shielding by phospholipids and bile salts. Charge shielding results in loss of repulsive electrostatic forces and consequently leads to aggregation. Aggregation was more pronounced in FeSSIF as it contains a higher concentration of phospholipids and bile salts. Moreover, lower pH favors protonation of amino groups and deprotonation of carboxylic moieties resulting in a comparatively more positive surface charge and, consequently, stronger binding of negatively charged compounds. Thus, tailoring the thiolated polymers toward a negative surface charge is likely advantageous when applying them to nanocarriers. Here, this was achieved using 6-MNA as an acidic S-protecting ligand. Attaching an increased amount of 6-MNA to thiolated polymers as achieved in previous studies^19,20^ likely leads to a further decrease of the ζ potential and could possibly prevent aggregation in applied media. The less-reactive and acidic ligands for S-protection such as thioglycolic acid could combine advantages of comparatively lower reactivity with increased stability of nanoparticles in biorelevant media.

### 4.3 Mucus Interaction of SLN Formulations

Within this study, the interaction between mucus and SLN formulations was investigated in terms of rheological interaction, SPT, mucus diffusion, and in terms of their ability to remain on an ex vivo mucosal tissue.

Strain sweep measurements provide proof for a stronger, and thus, covalent interaction between mucus and all SLN coated with thiolated polymers.^36^ In accordance with strong interaction, CS-Cys- and CS-Cys-Cys-coated SLN exhibited reduced diffusion in SPT experiments. Further, SPT could discriminate between CS-coated, CS-Cys-coated, and CS-Cys-Cys-coated SLN with respect to their percentage of hindered and immobilized particles. The rank order of particle–mucus interaction strength was derived as follows: CS-Cys > CS-Cys-Cys > CS > CS-Cys-MNA > plain.

Clearly, there is a discrepancy between the strong adhesion of CS-Cys-MNA in rheological experiments and low particle mucus interaction in SPT. Diffusion of CS-Cys-MNA-coated SLN in rear segments of a mucus layer additionally supports the results obtained from SPT. We hypothesized that CS-Cys-MNA as the only polymer exhibiting a negative surface charge has a lower affinity to the likewise negatively charged plain SLN. During covalent mucus interaction, the polymer might be stripped off and plain SLN dissociate from the coating material. This hypothesis was proven by labeling the coating material with FITC and subjecting SLN with likewise labeled coating material and lipid matrix to the rotating tube assay. Indeed, a distinctly different diffusion behavior of coating material and SLN could be observed. The FITC-labeled CS-Cys-MNA thus rather formed a mucoadhesive matrix from which plain SLN could diffuse into the mucus layer than serving as a coating agent. To achieve stronger interactions between the coating and SLN and thus to prevent dissociation, an ionic surfactant might be advantageous over nonionic Pluronic F127.

While mucoadhesive nanoparticles suffer from drawbacks where mucus permeating nanoparticles offer benefits and vice versa,^37^ S-protected thiolated polymers were able to provide both strong adhesion and efficient mucus diffusion by two different mechanisms. As shown in recent studies,^19,20^ decreased reactivity of l-cysteine as S-protecting ligand resulted in a higher diffusivity and deeper mucus diffusion while still showing stronger interaction than nonthiolated SLN. CS-Cys-MNA, on the other hand, served as a matrix from which SLN could diffuse into the mucus layer. As these formulations are both retained to a higher extent on the porcine intestinal mucosa, this study highlights the importance of combining mucoadhesive and mucus-penetrating properties for efficiently improving mucosal residence time.

## 5 Conclusions

Within this study, a less-reactive S-protecting ligand was introduced to a thiolated polymer using a thiol/disulfide-exchange reaction. CS-Cys, CS-Cys-MNA, and CS-Cys-Cys were successfully applied as coating materials to SLN and resulted in characteristic changes of the surface charge. A negative surface charge as induced by CS-Cys-MNA was advantageous in terms of stability in biorelevant media. Both S-protected thiolated polymers allowed strong interaction with mucus as well as diffusion of SLN into mucus. Consequently, CS-Cys-MNA-coated and CS-Cys-Cys-coated SLN were retained to a higher extent on porcine intestinal mucosa than all other formulations. Two different strategies to achieve a synergism of mucoadhesion and diffusion were enabled in this study: (1) application of l-cysteine as an S-protecting ligand to decrease the reactivity of the thiolated polymer and (2) application of the coating material with strong mucus interaction but a lower affinity to the plain particle allowing dissociation of the particle from the coating material.

## Supporting Information Available

^1^H NMR and FT-IR spectra of unmodified CS and target products; powder X-ray diffractograms of bulk compounds and SLN formulations; ζ potential after 30, 90, and 180 days of storage (PDF)

## Abbreviations Used

CS: chitosan
l-cysteine-HCl × H_2_O: l-cysteine hydrochloride monohydrate
CS-Cys: l-cysteine-modified thiolated chitosan
CS-Cys-MNA: 6-MNA-S-protected thiolated chitosan
CS-Cys-Cys: l-cysteine-S-protected thiolated chitosan
6,6′-DTNA: 6,6′-dithiodinicotinic acid
DTNB: 5,5′-dithiobis(2-nitrobenzoic acid)
DMF: *N*,*N*′-dimethylformamide
DMSO: dimethyl sulfoxide
DLS: dynamic light scattering
EDAC × HCl: 1-ethyl-3-(3′-dimethylaminopropyl)carbodiimide hydrochloride
EFTEM: electron filter transmission electron microscope
FaSSIF: fasted-state simulated intestinal fluid
FeSSIF: fed-state simulated intestinal fluid
FITC: fluorescein isothiocyanate
FT-IR: Fourier-transform infrared spectroscopy
HCl: hydrochloric acid
LBF: lipid-based formulations
LGR: Lumogen Red
MSD: mean-squared displacement
6-MNA: 6-mercaponicotinic acid
NHS: N-hydroxysuccinimide
PBS: phosphate-buffered saline
NMR: nuclear magnetic resonance spectroscopy
PXRD: powder X-ray diffraction
PDI: polydispersity index
KH_2_PO_4_: potassium dihydrogenphosphate
KCl: potassium chloride
NaCl: sodium chloride
SGF: simulated gastric fluid
SIF: simulated intestinal fluid
SPT: single-particle tracking
SLN: solid lipid nanoparticles
NaOH: sodium hydroxide
Na_2_HPO_4_: sodium phosphate monobasic
