# Supplementary material for: In Vitro Investigation of Thiolated Chitosan Derivatives as Mucoadhesive Coating Materials for Solid Lipid Nanoparticles
Source: Biomacromolecules. 2021 Aug 30;22(9):3980–91. doi: 10.1021/acs.biomac.1c00776 (PMC8441978; doi:10.1021/acs.biomac.1c00776)
Supplement: Supplementary file 1 — bm1c00776_si_001.pdf [file bm1c00776_si_001.pdf]

In Vitro Investigation of Thiolated Chitosan Derivatives as Mucoadhesive Coating Materials for Solid Lipid Nanoparticles

*Richard Wibel[1], Doris E. Braun[1], Laurenz Hämmerle[1], Arne M. Jörgensen[1], Patrick Knoll[1], Willi Salvenmoser[2], Christian Steinbring[1], and Andreas Bernkop-Schnürch[1]**

[1] Department of Pharmaceutical Technology, University of Innsbruck, Institute of Pharmacy, Center for Chemistry and Biomedicine, 6020 Innsbruck, Austria

[2] Department of Zoology, University of Innsbruck, Technikerstr. 25, 6020 Innsbruck, Austria

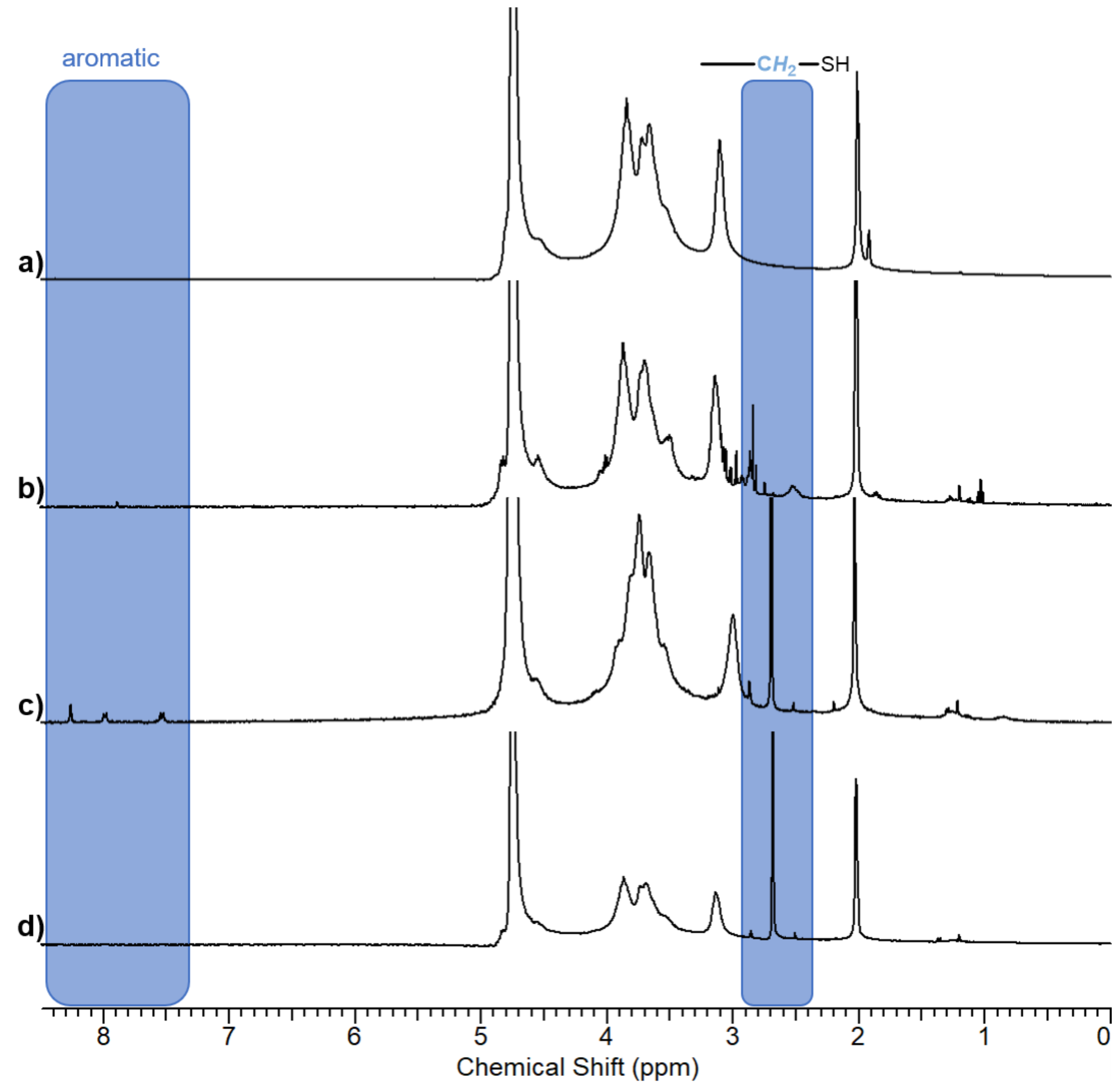

Figure S1. 400 MHz ^{1}H NMR spectra of a) CS, b) CS-Cys, c) CS-Cys-MNA, and d) CS-Cys-Cys recorded in D_2O + 1% acetic acid-d_4.

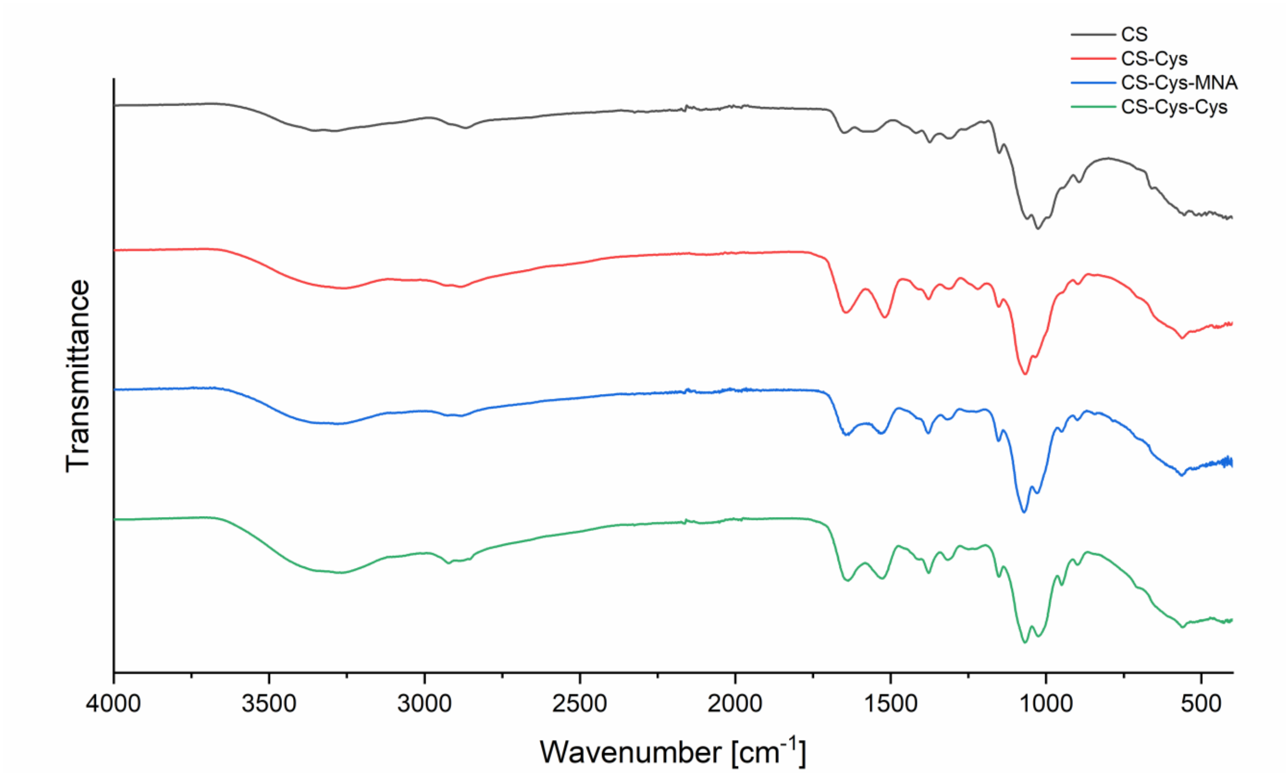

Figure S2. FT-IR spectra of unmodified CS (black), CS-Cys (red), CS-Cys-MNA (blue), and CS-Cys-Cys (green).

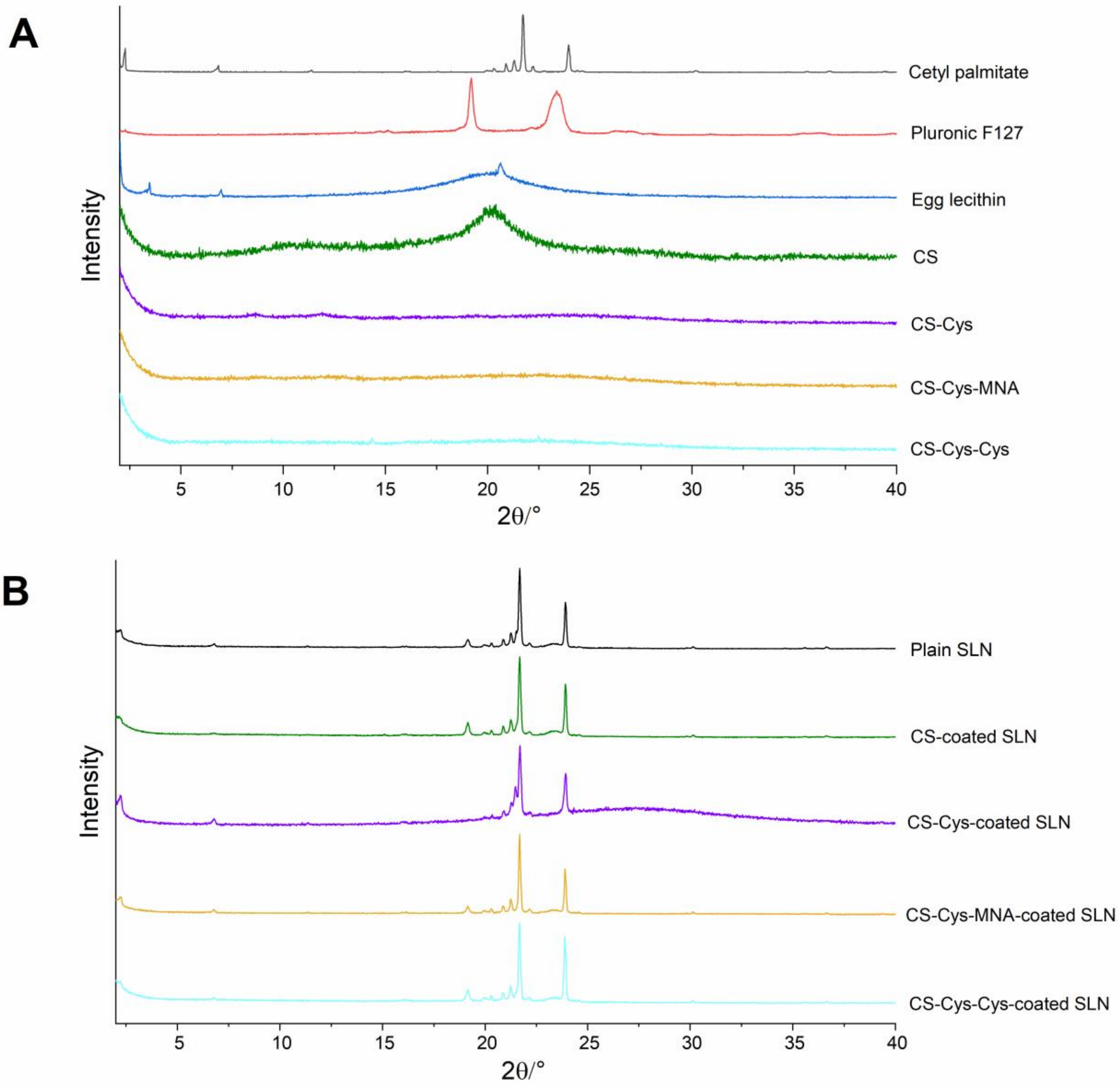

Figure S3. X-ray diffractograms of bulk compounds (A) and SLN formulations (B).

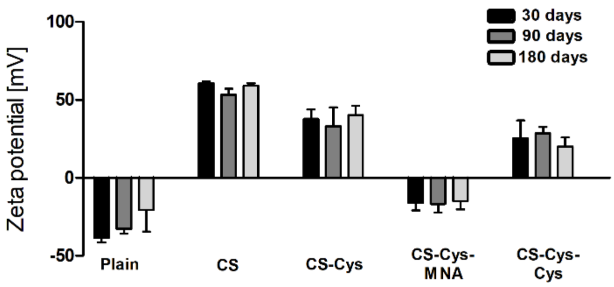

Figure S4. Zeta potential of SLN formulations after 30, 90, and 180 days of storage at 4°C (n ≥ 3).
